# Repurposing drugs in oncology (ReDO)—cimetidine as an anti-cancer agent

**DOI:** 10.3332/ecancer.2014.485

**Published:** 2014-11-26

**Authors:** Pan Pantziarka, Gauthier Bouche, Lydie Meheus, Vidula Sukhatme, Vikas P Sukhatme

**Affiliations:** 1 Anticancer Fund, Brussels, 1853 Strombeek-Bever, Belgium; 2 The George Pantziarka TP53 Trust, London KT1 2JP, UK; 3 GlobalCures, Inc; Newton MA 02459, USA; 4 Beth Israel Deaconess Medical Centre and Harvard Medical School, Boston, MA 02215, USA

**Keywords:** drug repurposing, cimetidine, immunostimulant, ReDO project

## Abstract

Cimetidine, the first H_2_ receptor antagonist in widespread clinical use, has anti-cancer properties that have been elucidated in a broad range of pre-clinical and clinical studies for a number of different cancer types. These data are summarised and discussed in relation to a number of distinct mechanisms of action. Based on the evidence presented, it is proposed that cimetidine would synergise with a range of other drugs, including existing chemotherapeutics, and that further exploration of the potential of cimetidine as an anti-cancer therapeutic is warranted. Furthermore, there is compelling evidence that cimetidine administration during the peri-operative period may provide a survival benefit in some cancers. A number of possible combinations with other drugs are discussed in the supplementary material accompanying this paper.

## Current usage

### Introduction

Cimetidine (CIM) is a histamine H_2_-receptor antagonist (H_2_RA), in the same class as ranitidine, famotidine, and nizatidine. By blocking the action of histamine on gastric parietal cells, the H_2_RAs are able to reduce the production of gastric acid, and indeed, CIM, the first drug in this class, was developed as a treatment for dyspepsia. In addition to dyspepsia, it is also used clinically for the treatment of peptic ulcers and for gastroesophageal reflux disease (GERD). The original trade name was Tagamet (GlaxoSmithKline), but the drug is now widely available as a generic. It is available as an over-the-counter drug in some countries, including the USA.

### Dosage

Oral CIM is available in tablet form and as a liquid suspension; it can also be used intravenously. Tablets are commonly available as 200 mg, 400 mg, and 800 mg doses. For the treatment of gastric or duodenal ulcer, the adult dosage varies between 800 mg and 1600 mg a day, either as single or divided doses throughout the day, for a period of 4–8 weeks. For reflux oesophagitis, the dose is 400 mg four times a day, for a period of 4–8 weeks. CIM is also used for maintenance therapy of gastric ulcers and short bowel syndrome at a daily dose of 400 mg, with a long-term treatment extending to greater than 10 years in some cases [1].

### Toxicity

CIM has low toxicity, with the most common side effects being headache, dizziness, diarrhoea, and rash. Rare side effects include gynaecomastia, reversible impotence (particularly reported in patients receiving very high doses, for example, in the treatment of Zollinger-Ellison Syndrome) and, very rarely, galactorrhoea. Rarely, CIM has also been associated with reversible leukopenia and thrombocytopenia, effects that may be particularly important to watch for in cancer patients who may be undergoing chemotherapy [2]. CIM is contraindicated during pregnancy. In children over the age of one, oral CIM may be used at a dose of 25–30 mg/kg body weight per day in divided doses. For children below the age of one, a dose of 20 mg/kg body weight per day in divided doses has been used [3].

### Pharmacokinetics

The bioavailability of CIM is 60–75%, with an elimination half-life of about 2–3 hours. Elimination is mainly via the kidneys, with excretion of the unchanged drug between 60% and 40% depending on the dose and method of administration [4]. Plasma concentrations peak at around one hour if taken without food, or after 2 hours with food. When taken without food, there is a second spike in plasma concentration after around 3 hours. Peak plasma concentration is barely affected by food, with values of 1.18 µg/ml and 1.09 µg/ml, respectively, after an oral dose of 200 mg [5]. Plasma concentrations during continuous treatment with 1.0 g/day were above 1.0 µg/ml for 9 out of 24 hours [4].

CIM is an inhibitor of cytochrome P450, through multiple enzymes (including CYP1A2, CYP2D6, and CYP3A, CYP3A3/A4, CYP2C9, and CYP2C18), which may have a significant impact on the metabolism of a wide range of drugs [6, 7]. In terms of chemotherapy, there is evidence, for example, that concomitant use of CIM and epirubicin can raise the area under the curve (AUC) of both the parent compound and the main metabolites significantly in patients with breast cancer [8]. While this suggests caution in clinical use of CIM for cancer patients, there is also a potential for therapeutic benefit if this effect is deliberately utilised to increase the plasma level or delay clearance of drugs being used for anti-cancer purposes, such as the benzimidazole anti-helminthic mebendazole [9].

### Other relevant interactions

CIM has been investigated as an agent to reduce side effects of some current treatments. Examples include the use of oral CIM (800 mg daily) to reduce side effects radiotherapy of the para-aortic lymph nodes in the treatment of cervical cancer [10], and in the prevention of vinorelbine-induced phlebitis using intravenous CIM [11]. Nephrotoxicity is a serious side effect of cisplatin, an adverse effect mediated by the organic cation transporter 2 (OCT2) regulated uptake of the drug in proximal tubules. CIM is a potent inhibitor of OCT2, and *in vitro* investigations showed that it did not alter the uptake or cytotoxicity of cisplatin in an ovarian cancer cell line (IGROV-1) expressing high levels of OCT2. Subsequent tests in a mouse model showed no diminution of the anti-tumour effect of cisplatin. In patients with head and neck cancer, concurrent CIM (800 mg twice a day) did not alter the exposure to unbound cisplatin, a proxy measure of anti-tumour activity [12].

## Pre-clinical evidence in cancer—*in vitro* and *in vivo*

Early interest in the potential anti-cancer action of CIM was aroused by investigations into the relationship between histamine levels and cancer, particularly by the finding that histamine levels were increased in distant non-cancer tissues in tumour-bearing mice [13, 14] and decreased in plasma, a result confirmed in humans [15]. An early investigation suggested that a histamine H_2_ receptor agonist stimulated cellular proliferation in dimethylhydrazine-induced colonic carcinoma in rats, an effect reversed by CIM and another H_2_RA (metiamide), and that an H_1_ receptor antagonist had no effect [16]. Initial results also suggested that treatment with metiamide could slow tumour growth and increase survival in sarcoma-bearing mice [17].

Subsequent *in vivo* results were also obtained with CIM in a number of rat and mouse models by different research groups, with attention focused in particular on immunomodulatory mechanisms [18–20]. Other early *in vivo* results indicated that CIM could enhance the cytotoxic effect of cyclophosphamide in male DBA2 mice injected with P-388 leukaemia cells, significantly increasing survival time [21]. However, there were issues with study replication, and it was reported by Hannant *et al* that *in vivo* results from other groups could not be reproduced [22]. Similarly, the potentiation of the cytotoxic effect of cyclophosphamide could not be replicated with a different mouse model, although a potentiation of the anti-tumour effect of razoxane was reported [23].

One possible explanation for these mixed results was suggested by the results in immunocompetent and immunosuppressed DBA2 mice, which showed that CIM reversed the accelerated growth of implanted tumours in immunosuppressed mice known to have higher levels of suppressor cell activity but had no effect on normal mice (though in one of three experiments CIM treatment increased tumour growth in normal mice, a result possibly due to atypically slow tumour growth in this experiment) [24].

The effect of CIM, often in combination with other agents, particularly immunomodulators such as interferon or IL2, has continued to be explored in numerous *in vivo* studies in a range of cancer types, including melanoma [25], ovarian cancer [26], colorectal cancer [27], gastric tumours [28], pancreatic cancer [29], lung cancer [30], and gliomas [31] in the years since the early 1980s. It is beyond the scope of this paper to fully summarise this wide range of studies, only a few of which have been referenced here, particularly as there is a comparable range of clinical work being carried out and which is summarised in the next section. However, it should be noted that it is the clinical evidence that is of primary interest from the point of view of drug repurposing [32].

## Human data in cancer

One of the earliest references to an anti-cancer effect of CIM comes from a report of two cases published in *Lancet* in 1979 [33]. In both cases, patients with metastatic disease had displayed some tumour regression following treatment with CIM. This, and the subsequent flurry of animal results that focused primarily on the putative immunomodulatory role of CIM, initiated many clinical studies in the use of CIM in oncology. A full survey is beyond the scope of this paper, but the main results are summarised below, listed for those cancers for which there is the highest level of clinical evidence.

### Colorectal cancer

Based on earlier *in vitro* and *in vivo* results [27], Adams and coworkers investigated the use of perioperative CIM in patients undergoing surgical resection of colorectal cancer. Control patients showed significant falls in lymphocyte proliferation and cell-mediated immunity. In contrast, patients treated with oral CIM at a dose of 400 mg twice a day for a minimum of 5 preoperative days, then intravenously for 2 post-operative days, showed no significant falls in either lymphocyte proliferation or cell-mediated immunity, indicating that CIM helped reduce post-operative immunosuppression following resection [34]. There was some indication that this difference could provide some clinical benefit in a follow-up that looked at survival at 3 years in two subsequent reports, which showed that with a median follow-up at 30 months, the calculated 3-year survival was 93% for CIM-treated patients and 59% for controls [35, 36].

In a randomised, blinded trial, Svendsen *et al* treated 192 patients with oral CIM, at a dose of 400 mg twice a day, following surgery (resection or exploratory) for colon (123 patients) or rectal (69 patients) cancer [37]. CIM treatment commenced in the first 3 weeks following surgery and continued for 2 years. The primary end point was cancer-specific mortality. In patients treated with curative intent (148 patients), there was no difference in this end point between the treatment and control arms. However, on stratification, the curatively treated patients with Dukes C disease tended towards lower cancer-specific mortality, though this did not reach statistical significance (29% reduction, 90% confidence interval 2–57%, p = 0.11 log-rank test). There were no differences between groups in the non-curatively treated patients.

In another double-blinded trial of preoperative CIM in Australia, 112 colorectal cancer patients were randomised to low-dose (400 mg twice a day), high-dose (800 mg twice a day), or placebo arms [38]. Treatment was given for five days prior to surgical excision. Kaplan-Meier survival analysis showed there were no significant differences between treatment groups, although there was a trend towards survival advantage in the high-dose CIM (800 mg) group (p = 0.20, log-rank test). However, stratification by replication error (RER) positive or negative tumours showed a statistically significant difference between the high-dose CIM group and the placebo group (p = 0.04, log-rank test) for patients with RER negative tumours.

An unblinded randomised multi-centre trial in Japan reported on long-term survival of colorectal cancer patients treated post-operatively with oral CIM, at a dose of 800 mg daily [39]. A total of 72 patients with colorectal cancer and a primary tumour of T2 or T3 were enrolled, after exclusion of patients who had previously been treated with chemotherapy, radiotherapy, or immunotherapy or who had multiple cancers or severe complications. Of these 72 patients, two did not undergo curative resection, three did not receive adequate drug administration, and three whose disease stage was considered inappropriate for the trial were further considered ineligible and were excluded from the analysis. The remaining 64 patients were randomly allocated, and there were none lost to follow-up. All patients underwent curative resection and then received 200 mg per day of oral 5-U for one year. The treatment group of 34 patients additionally received 800 mg per day of CIM for the 1-year treatment period. In both groups, treatment started 2 weeks post-surgery. Mean follow-up was 10.7 years and showed that the 10-year survival rate of the treatment group was 84.6%, whereas that of a control group was 49.8% (p < 0.0001).

A trial of oral CIM during the perioperative period in 49 patients suffering from gastrointestinal tumours investigated the effect on the immune response, including 19 patients suffering from colorectal cancer [40]. Patients were randomised to perioperative CIM (24 patients) or control (25 patients), with an equal number of colorectal cancer patients (19) in each arm. The patients in the treatment group received 400 mg of oral CIM three times a day for 7 days prior and up to the day of surgery, and then intravenous CIM at 600 mg twice a day during and post-surgery for 10 days. The control arm received standard of care without CIM. The primary end points were measurement of immune status, including peripheral blood lymphocytes, natural killer (NK) cells and tumour-infiltrating lymphocytes (TIL). No clinical outcomes were assessed. In comparison with blood counts from healthy individuals, both treatment and control arms showed decline in the proportion of total T cells, T helper cells, and NK cells. These changes were reversed in the patients in the CIM arm, who showed significantly higher counts on the 10th post-operative day than controls. Also significant was the difference in the number of patients showing increases in TIL response: 68% (17/25) of the patients in the treatment group had significant TIL responses, and only 25% (6/24) of the cases had discernible TIL responses (p < 0.01).

A 2012 Cochrane Review of H_2_RAs as adjuvant treatments for resected colorectal cancer pooled data from six randomised clinical trials, five of which utilised CIM and one used ranitidine. The review found a trend towards improved survival when H_2_RAs were utilised as adjuvant therapy in patients having curative-intent surgery for colorectal cancer (HR 0.70; 95% CI 0.48-1.03, P = 0.07). However, analysis of the five CIM trials (with pooled data for 421 patients) found a statistically significant improvement in overall survival (HR 0.53; 95% CI 0.32 to 0.87) [41]. Overall the authors concluded that: *cimetidine appears to confer a survival benefit when given as an adjunct to curative surgical resection of colorectal cancers*.

### Melanoma

The earliest clinical evidence of an effect of the CIM on melanoma was in a series of three cases of recurrent malignant melanoma being treated with coumarin (at a dose of 100 mg per day). Oral CIM was started at a dose of 1000 mg per day when these patients were no longer responding to coumarin treatment. In these cases there was rapid regression of multiple lesions and a corresponding and long-lasting improvement in physical condition. In one further case, recurrent disease was treated with a lower dose of coumarin (25 mg) and CIM (1000 mg), but the disease progressed rapidly, and the patient died shortly after. This patient had not previously been treated with coumarin [42].

A similar pattern was recorded in a series of six melanoma patients, five of whom had disseminated disease, treated with human leukocyte interferon-alpha, with little evidence of effect. The addition of CIM after a period of 6–8 weeks led to a remarkable change, with complete remissions seen in two patients, a partial remission in one, and disease stabilisation in another [43]. The same authors subsequently reported on 20 patients who had also been treated with interferon-alpha with no objective responses; the subsequent introduction of CIM led to six objective responses, including five complete regressions and one extensive partial regression, and three cases of prolonged disease stabilisation [44].

At that time, the standard of care treatment for metastatic melanoma was treatment with dacarbazine or nitrosoureas, with an objective response rate of around 15% and median overall survival of 4 months [45].

A series of seven Phase II studies, in 191 patients, on the use of recombinant interferon-alpha 2a (rIFN-2a), alone and in combination with other agents, for the treatment of disseminated malignant melanoma was carried out by Creagan *et al*. One of these studies included oral CIM, at a dose of 1200 mg per day (300 mg QID), as an immunostimulant [46]. The response rate for these diverse studies ranged from 0% to 23%, with an aggregate response rate of 14% (27 of 191 patients), and median survival time was 6 months [47]. The best responses, at 23%, were for rIFN-2a monotherapy or rIFN-2a with CIM, suggesting no additional benefit for CIM, although there was a lower level of toxicity in the CIM group.

A later Phase II study, by a different group, looked at the combination of interferon-alpha 2b, IL-2, and cisplatin in metastatic melanoma in a group of 87 patients, with and without oral CIM. An overall response rate of 27% was achieved in the 82 patients evaluable for response, with median response duration of 7 months and median survival of 10.1 months. There were no significant differences between the CIM and non-CIM arms of the study [48].

It is possible that the difference in outcomes between the earlier trials and the more disappointing later trials may be related to the different formulations of interferon used. The form of interferon used by Flodgren *et al* was human leukocyte derived (HuIFN-alpha (Le)) [43, 44], whereas the work of Creagan *et al* [47] and Schmidt *et al* [48] used recombinant interferon 2 alpha. Human-derived interferons contain a wider range of alpha interferon subtypes than recombinant interferons, which are normally restricted to alpha 2a or 2b, and there is some anecdotal evidence that recombinant interferons may not be as effective in stopping tumour development [49]. It may be hypothesised that CIM interacts with some additional alpha interferon subtypes to potentiate the effect and to improve response to treatment, as shown in the earlier trials.

Investigation of CIM as a monotherapy in metastatic melanoma was also investigated in two small Phase II trials, by different groups. In the first trial, 19 previously untreated patients were treated with oral CIM at 1200 mg (300 mg QID). Objective responses were observed in three (19%) patients, including one long-lasting (16+ months) complete response, one near-complete response (21+ months), and one partial response (7 months). Overall median time to progression was 1.4 months, and overall survival was 6 months [45]. A later trial of CIM monotherapy involved 15 treatment-naïve patients who were treated with high-dose CIM (600 mg QID), although three patients experienced stable disease for 2–4 months. There were no objective responses recorded, and median survival time was 5.3 months, suggesting little significant activity as a monotherapy in this group of patients, although toxicity was negligible even at this high dose [50].

### Gastric

Early concerns were raised that the treatment of duodenal ulcers with CIM might alleviate the symptoms of gastric carcinoma, thereby masking disease progression [51], or that CIM itself may be carcinogenic and increase the risk of gastric cancers [52]. However, long-term post-marketing surveillance has shown no such association [53].

A report from Denmark assessed overall survival of gastric cancer patients treated with oral CIM at 800 mg per day (400 mg BID) for 2 years. In this double-blinded study, 181 patients were randomised to CIM or placebo immediately after surgery or the decision not to operate. Median survival in the CIM group was 450 days and 316 days in the placebo group, a statistically significant result (p = 0.02, log-rank test). Relative survival rates (CIM/placebo) were 45%/28% at 1 year, 22%/13% at 2 years, 13%/7% at 3 years, 9%/3% at 4 years, and 2%/0% at 5 years [54].

However, a larger randomised, double-blinded trial in the UK, involving 442 patients, did not find a positive effect of oral CIM [55]. Patients were randomised to either low-dose (400 mg, BID) or high-dose (800 mg, BID) CIM or placebo until tumour progression, recurrence, or death. The median survival for patients receiving CIM was 13 months (95% confidence interval, 9–16 months) and 11 months for the placebo arm (95% confidence interval, 9–14 months), a result that did not reach statistical significance. Within the CIM arms, median survival for the high-dose group was 13 months (95% CI 7–20 months), and 13 months (95% CI 8–18 months) for the low-dose. The 5-year survival was 21% for those randomised to CIM compared with 18% in the placebo arm, again a result that did not achieve statistical significance.

### Renal cell carcinoma

The earliest clinical evidence that CIM might have some effect on renal cell carcinoma (RCC) was from a small trial that looked at the combination of CIM and coumarin in 45 patients suffering from metastatic RCC [56]. Patients were treated with coumarin, 100 mg orally daily; on day 15 of treatment, oral CIM was started at a dose of 1200 mg (300 mg QID), and treatment with both drugs was continued until disease progression. Of 42 evaluable patients, there were three complete responses (CR) and eleven partial responses (PR), giving an objective response rate of 33.3%. Twelve patients exhibited stable disease (SD). The median duration of the PR group was 5 months (in the range 4–21+ months), while the median duration of the SD group was 7.3 months (in the range 4–16.5+ months). There were no reported toxicities with the treatment. Subgroup analysis showed that there were no objective responses in the 14 patients who had not undergone nephrectomy, whereas the fourteen objective responses occurred in the 31 patients who had undergone nephrectomy.

A number of subsequent studies were unable to reproduce this encouraging result. A similar protocol was used in a three-centre Phase II trial that enrolled 31 patients, the majority of whom (84%) had been nephrectomised [57]. Whereas the original study used CIM at 300 mg four times a day, this trial used a dose of 400 mg three times a day, in all other respects the protocol was the same. Of the 31 patients treated, only two (6.5%) showed a PR of 63 weeks and 73 weeks. Both patients experienced regression of pulmonary metastases. Five patients experienced SD (in the range 28–45+ weeks). Similarly, another small study used a protocol identical to the original to treat 25 patients, 21 of whom had been nephrectomised. Here there were no objective responses recorded, although five patients experienced SD for more than 3 months. One possible explanation for the disparity may be explained by the better performance status and lower tumour burden of the patients in the original study [58].

CIM has also been investigated in combination with human lymphoblastoid interferon-alpha (LIFN-a) in RCC. A total of 37 patients with advanced RCC were treated between 1982 and 1995 in Japan, of whom 21 patients had metastatic disease at presentation, and 15 had recurrence after nephrectomy. LIFN-a was administered intramuscularly at 5 million units (MU) daily for 5 to 7 days a week for at least 8 weeks, and CIM was administered orally at 200 mg QID [59]. Treatment resulted in an objective response rate of 41%, with a CR in seven patients and a PR in eight. Additionally, 12 patients exhibited SD. Patients with lung metastases showed the best response to therapy. The 5-year survival rates for patients with and without response and overall were 74%, 20%, and 41%, respectively. Histopathologically, high-grade tumours had a better response to combined therapy than did low-grade tumours.

A subsequent Phase III trial by the same group compared treatment of LIFN-a alone with LIFN-a and CIM, with 36 patients recruited to LIFN-a alone and 35 patients to combined LIFN-a and CIM. Intention-to-treat analysis showed one CR, four patients with PR, 16 with SD, and 12 with progressive disease (PD) among the 36, with an overall response rate of 13.9%. Of the 35 patients in the LIFN-a and CIM arm, there were two cases of CR, 8 patients with PR, 13 with SD and 11 with PD, yielding a response rate of 28.6% (P = 0.13). Time to progression ranged from 9 to 845 days (median 112 days) in the LIFN-a group, and from 31 to 1,568 days (median 125 days) in the LIFN-a plus CIM group (P = 0.87) [60]. While tending towards improved response, the authors concluded that the addition of CIM to LIFN-a did not significantly improve the response rate compared to LIFN-a alone.

Despite this conclusion, interest in the combination of interferon and CIM for advanced RCC continues with the addition of other agents. For example, the combination of LIFN-a, CIM, the COX-2 inhibitor meloxicam and the angiotensin II receptor antagonist candesartan was investigated in the Phase II (I-CCA) trial involving 51 patients, of whom 37 (73%) had received prior nephrectomy [61]. Patients received 3–6 MU of LIFN-a, 400 mg CIM BID, 10 mg meloxicam daily and 4 mg candesartan daily. Initially the angiotensin converting enzyme (ACE) inhibitor perindopril erbumine was used, but as this caused a persistent cough in some patients, it was replaced with candesartan. CR was observed in four patients (8%) and PR in seven (14%), giving an overall response rate of 22%. None of the four CR patients relapsed during the 16–81 month follow-up. Of the remaining patients, 24 patients (45%) had SD for at least 6 months, yielding a clinical benefit in 67% of patients, with no grade 3/4 toxicities observed. The median progression-free survival and overall survival were 12 and 30 months, respectively. These results were sufficient for the authors of the study to conclude that the therapy was a potential first-line treatment for advanced RCC that needed to be confirmed in a large international Phase III trial.

The use of high-dose CIM as a single agent in metastatic RCC has also been the subject of a Phase II clinical trial involving 42 patients in the United States. Patients, of whom 38 were evaluable, were treated with 600 mg CIM QID. Two patients showed CR, one of 26 months and one of 33+ months, yielding an objective response rate of 5.3%. There were no cases of PR and four cases of SD (duration in the range 3–9+ months). Both patients with CR had experienced prior nephrectomy [62]. At this relatively high dose, toxicity was minimal, with one case of mild leukopenia reported.

### Other cancers

In addition to the clinical investigations in colorectal and gastric cancer, melanoma, and RCC, there have been a few clinical studies in other cancer types. An investigation of a possible correlation between preoperative CIM and measures of tumour cell proliferation (Ki-67 staining) in breast cancer found no association [63].

In pancreatic cancer, there is a recently published case report of activity of the anti-angiogenic agent TL-118, which includes CIM as one of four drugs that make up the combination agent [64]. In this case report, a 75-year old woman with radiologically confirmed inoperable pancreatic cancer has been treated with TL-118 and gemcitabine and has shown a long-lasting (16 months) progression-free survival. Treatment interruption correlated with an increase of tumour marker CA 19-9, and resumption of treatment reduced levels of this marker.

In metastatic prostate cancer, an early trial looked at the combination of CIM and coumarin, using the same protocol as that for melanoma and RCC [65]. While no objective responses were reported in the fourteen patients in the trial, three patients experienced significant reduction in pain from bone metastases and decreased analgesic use that persisted until disease progression at 3, 5.5+, and 9 months.

A small trial in 28 advanced serous ovarian carcinoma patients found that standard platinum-based chemotherapies augmented with CIM, at a dose of 800 mg/day, commencing 2 weeks before surgery and continuing synchronously with chemotherapy, showed statistically significant improvements in overall survival compared to platinum-based chemotherapy alone [66].

The use of oral CIM in the treatment of Kaposi’s Sarcoma in patients with AIDS was investigated in eight patients with progressive disease (PD) [67]. CIM was given orally at a dose of 300 mg QID, rising to 600 mg QID if there was no response within one month. Of the eight patients evaluated for response, one showed a complete remission of 7+ months, one patient had a partial response of 8 months and one showed a mixed response of initial regression followed by PD. The other five patients all showed PD. No patients reported toxicity, and several reported symptomatic improvements.

## Clinical trials

TL-118 is a novel drug combination produced by Tiltan Pharma Ltd, Israel. Designed as a multi-targeted anti-angiogenic agent, the four drugs that make up the combination are: CIM, low-dose cyclophosphamide, diclofenac, and sulfasalazine. TL-118 is formulated as an oral suspension and is designed to be taken by patients at home rather than administered in a clinical setting. Currently, there are three Phase II clinical trials of TL-118:

NCT01509911 is an international multi-centre trial in metastatic pancreatic cancer for patients starting gemcitabine treatment. The primary outcome is the disease control rate after 16 weeks of treatment.

NCT01659502 is a single centre study in pancreatic cancer. The primary outcome is a clinical benefit measurement (a composite score based on pain, performance status, and weight) in a 2-year time frame.

NCT00684970 is a multi-centre, single-country trial designated as a Phase IIB trial for metastatic castration-resistant prostate cancer. The primary end point is progression-free survival from 24 weeks after commencement of treatment up to 3 years. Secondary end points include overall survival, time to prostate-specific antigen (PSA) progression, PSA response, and pain response in evaluable patients.

A randomised, double-blinded Phase II trial in Australia and New Zealand (ACTRN12609000769280) is investigating the perioperative use of CIM in patients with colorectal cancer treated with curative resection. The dose is 800 mg tablets twice daily for 5 weeks, starting a week before surgery. The primary outcome is 2-year disease-free survival, with additional subgroup analysis of patients with positive tumour staining for sialyl Lewis antigens. Secondary end points include longer-term disease-free survival, overall survival, and duration of postoperative inflammatory cytokine elevation, assessed as the time that plasma concentrations of each cytokine (TNF, IL-1B, IL-6, IL-8) are elevated above pre-treatment baseline. Recruitment to the trial is complete, and 45% of the cohort have rectal cancer [68].

## Mechanism of action

The anti-tumour action of CIM has been shown to be due to four distinct mechanisms:

- Anti-proliferative action on cancer cells
- Immunomodulatory effects
- Effects on cell adhesion
- Anti-angiogenic action

### Cancer cell proliferation

It has been shown, *in vitro* and *in vivo*, that multiple tumour types express the histamine-synthesising enzyme, L-histidine decarboxylase (HDC) and that tumours can secrete high levels of histamine in a paracrine and/or autocrine fashion. Histamine is highly pleiotropic, with multiple functions involving inflammatory immune response, gastric acid secretion, and action as a neurotransmitter. These diverse physiological actions are mediated by four histamine receptors, of which H_2_ and H_4_ are implicated in cancer cell proliferation, invasion, and angiogenesis [69, 70].

A direct effect on cancer cell proliferation has been shown in two xenograft experiments in which exogenous histamine increased tumour growth in C170 and LIM2412 human colorectal cell lines implanted in Balb/c *nu/nu* mice, an effect that was reversed by oral CIM but not by the H_1_RA diphenhydramine [27]. Similar results have been shown in gastric cancer, with locally applied histamine increasing the proliferation of implanted MKN45G xenografts in nude mice, an effect abrogated by CIM [71]. *In vitro* analysis in both colorectal and gastric cancer cell lines showed that the dose-dependent increase in cellular proliferation induced by histamine was associated with an accumulation of cyclic adenosine monophosphate (cAMP) [27, 71].

However, there is also some evidence to suggest that some anti-proliferative effects of CIM may not be entirely related to generic activity as an H_2_RA. A comparison of the anti-proliferative effect of different H_2_RAs in gastric cancer cell lines showed that CIM significantly reversed histamine-stimulated proliferation in a dose-dependent manner, ranitidine had a lesser effect and famotidine showed no effect [72]. This suggests either that there is something specific about the binding of CIM to the H_2_ receptor or else there are additional off-target effects of CIM action. For example, there is also some evidence CIM can cause apoptosis in the Caco-2 human colorectal cancer cell line independent of its action as an H_2_RA [73]. Similarly, while CIM synergised with a novel phospho-valproic acid to inhibit pancreatic tumour growth in mouse models, another H_2_RA, ranitidine, showed no such activity [74].

### Immunomodulation

Histamine has multiple effects on both innate and adaptive immune responses, mediated by the four histamine receptors (H_1_–H_4_). In relation to cancer, histamine is associated with an immunosuppressive tumour microenvironment, including an increase in CD4+CD25+ regulatory T cell (Treg) activity, reduced antigen-presenting activity of dendritic cells (DC), reduced NK-cell activity and increased myeloid-derived suppressor cell (MDSC) activity [75–77].

In particular, histamine binding to the H_2_ receptor is associated with suppression of IL-12 and stimulation of IL-10 secretion and is implicated with a shift in Th1/Th2 balance toward Th2-dominance of the immune response. This effect was reversed by CIM in human PBMC [78]. Similarly, in an HDC knock-out mouse model, animals inoculated subcutaneously with the LM2 murine breast cancer cell line showed slower tumour growth than in HDC wild-type mice. The knock-out mice, lacking endogenous histamine, showed a predominance of Th1 cytokines and a lower level of Foxp3 (associated with CD4+CD25+ Tregs) expression compared to wild-type tumour-bearing mice [79].

In addition to Treg cells, the other key drivers of the immunosuppressive tumour microenvironment are MDSC cells, involved in extensive cross-talk with Tregs in promoting T-cell dysfunction and in skewing the immune response towards Th2 [80]. MDSCs express H_1_–H_3_ receptors, and there is *in vitro* and *in vivo* evidence that blockade of H_1_ (using the H_1_RA cetirizine) or H_2_ (using CIM), can reverse the immunosuppressive action of these cells [77]. For example, the addition of CIM reduced the tumour burden in a B16 melanoma mouse model [77] and in a mouse model of 3LL lung tumour [30].

CIM has also been shown to increase the *in vitro* antigen-presenting activity of monocyte-derived DC, in advanced colorectal cancer patients compared to controls [81]. An increase in NK activity compared to non-CIM-treated controls has also been noted in cardiopulmonary bypass surgery [82].

Additionally, perioperative CIM has been shown to reverse the inhibition of lymphocyte proliferation induced by histamine and to increase the number of TIL in colorectal and gastric cancer patients [36, 40, 76]. Increased TIL was associated with prognostic significance in these trials, and is also considered significant in a range of other cancer types, including breast, ovarian, brain, and head and neck cancers.

### Cell adhesion

CIM has been shown to have an inhibitory effect on cancer cell adhesion to endothelial cells independent of its H_2_RA activity. Using a monolayer cell adhesion assay the adhesion of HT-29 colorectal cancer cells to human umbilical vein endothelial cells was investigated for CIM and two other H_2_RAs (famotidine and ranitidine). Where CIM inhibited adhesion in a dose-dependent manner, the other H_2_RAs had no effect. In a nude mouse model, CIM dose-dependently reduced the incidence of HT-29 liver metastases, suppressing it completely at the highest dose (200 mg/kg/day) [83]. The effect on cell adhesion was mediated by the interaction between tumour sialyl Lewis antigens and E-selectin expressed on the endothelium.

Subsequent investigation has shown that there is a positive correlation between response to CIM treatment in colorectal cancer patients and high expression levels of sialyl Lewis-X and sialyl Lewis-A [39]. The reported 10-year cumulative survival rate of the CIM group with higher staining of sialyl Lewis-X in tumours was 95.5%, whereas that of a control group was 35.1% (P = 0.0001).

In addition to colorectal cancer, the inhibitory effect on cell adhesion has been demonstrated for other cancers, including breast [84], salivary gland tumours [85], gastric cancer [86] and glioblastoma [31].

### Angiogenesis

The final mechanism of action that has been investigated in relation to the anti-cancer action of CIM is the effect it has on tumour neo-angiogenesis. Ghosh *et al* investigated the role of histamine in the production of vascular endothelial growth factor (VEGF) in carrageenin-induced granulation tissue in rats, and found that it was mediated by the H_2_ receptor, and that the upregulation of VEGF induced by histamine was reversed by CIM [87]. A study comparing CIM and roxatidine (another H_2_RA), found that both drugs strongly reduced colon 38 tumour implants in C57BL /6 mice syngeneic mice, and that this inhibition was related to reduced expression of VEGF and reduced micro-vessel density in the implanted tumours [88]. Additionally, there is also evidence that the anti-angiogenic effect of CIM administration may also be related to a reduced expression of platelet-derived endothelial growth factor (PDECGF), as well as VEGF, in mouse and rat models of bladder cancer [89].

Mechanistically, it has been suggested that VEGF expression is increased by histamine via the activation of the cyclooxygenase-2 (COX-2) pathway in colorectal cancer cell lines, a process mediated by the H_2_ and H_4_ receptors [90]. This process was disrupted by the H_2_RA zolantidine and H_4_RA JNJ 7777120. In contrast, CIM showed no effect on VEGF expression in an *in vitro* endothelial cell model of angiogenesis [91].

## Our take

### Next steps

The abundance of clinical evidence shows that CIM has demonstrable therapeutic effects in a range of cancers, particularly cancers of the gastrointestinal tract, RCC, and melanoma (summarised in Table 1). There is also evidence, both *in vitro* and *in vivo*, that these effects are most likely related to well-documented immunomodulatory effects. Furthermore, the evidence indicates that these effects may extend beyond the direct effect on the H_2_ histamine receptor and that CIM has off-target effects which are not shared by other H_2_RAs. We can hypothesise, therefore, that a portion of the variability of response to CIM reported in different clinical trials may be explained by the degree of variability of immune function in cancer patients. In common with other immunotherapeutic agents, this suggests that CIM may be more efficacious in patients with lower tumour burden and higher immune function, and in cancers with a greater antigenic potential. Indeed, an explanation proffered for the differences in response reported by different clinical trials in RCC was the better performance status (related to tumour burden) in patients in early trials compared to the poorer response reported in later trials [58].

This suggests that, in general, CIM should not be used as a single agent in an adjuvant setting, or in patients with large tumour burden or in cancer types which are known not to respond well to immunotherapeutic intervention. However, with that proviso, there is still considerable scope for clinical investigation of CIM as an immunostimulant, with a possible anti-metastatic action, in a range of cancer types.

In particular, one window of opportunity exists in using CIM to address the issue of post-operative immunosuppression. It is known that surgical resection, a mainstay of cancer treatment for many forms of the disease, causes a post-surgical immune suppression that may be associated with an increased risk of recurrence or metastatic spread [92, 93]. There is already strong evidence, summarised in a Cochrane Review, that *perioperative* CIM is associated with reduced immunosuppression and a lower risk of disease recurrence in the curative resection of colorectal cancer [41]. Moreover, the increased risk of post-surgical recurrence exists in other forms of cancer, including breast, lung, head and neck, and osteosarcoma. In breast cancer, for example, the perioperative use of the non-steroidal anti-inflammatory drug (NSAID) ketorolac is being investigated as potential agent to improve survival following mastectomy [94]. CIM is of potential benefit in these other cancers, in addition to the established benefit in colorectal cancer.

Given the evidence that *perioperative* CIM reduces post-surgical immunosuppression, it is suggested that there is a need for clinical trials to establish whether it may be of benefit, in terms of overall survival, in the following cancer types:

- Colorectal
- Breast
- Non-small Cell Lung Cancer
- Osteosarcoma
- Ovarian
- Pancreatic

It may be critical in these trials to start CIM treatment in the days immediately prior to surgery and continue for a number of weeks following, indeed it is worth noting that the most impressive clinical trial data show a dramatically improved survival for colorectal patients treated with oral CIM (800 mg/day) and oral 5-FU (200 mg/day)—the CIM-treated group had 10-year survival of 84.6% versus 49.8% for the 5-FU-only group [39]. Additionally, the investigation into the combined perioperative use of CIM and diclofenac/ketorolac warrants attention.

There is evidence that an immunological adjuvant may of benefit in a wide range of cancers, including some of those in which CIM has already shown some clinical benefit:

- Melanoma
- RCC
- Gastric cancer
- Glioblastoma

It is suggested that CIM be investigated as an adjuvant to the existing standard of care therapies in these diseases.

Given the primary putative mechanisms of action—the strongest evidence is for effects on immunity and cell adhesion—there are a number of additional agents that warrant investigation for synergy with CIM, some of which are listed in the supplementary material accompanying this paper.

### New protocols

It is instructive to review the ongoing clinical trials of CIM as an anti-cancer agent as they serve as useful templates for future investigations. The Australian trial of perioperative CIM in colorectal cancer (ACTRN12609000769280) builds directly on a number of similar earlier trials, which are effectively summarised in a Cochrane Review [41]. It is to be hoped that a positive result in this trial will focus attention once more on the potential of CIM to positively affect overall survival. Of note a recent analysis of the long-term results of the EORTC 22921 trial found that adjuvant fluorouracil-based chemotherapy after preoperative radiotherapy (with or without chemotherapy) does not affect disease-free or overall survival [95], suggesting that this is an indication where progress is urgently needed and where CIM already has shown strong clinical evidence of effect.

However, using CIM to address post-surgical immune suppression is not the only possible model of use. The other clinical trials on-going combine CIM with a number of other low-cost agents to form the novel drug combination TL-118. In this model of use, it is the combination of multiple repurposed drugs, with similar low toxicity and low costs, which together form effective and novel treatment options. In this manner, we can create multi-targeted protocols which pose minimal risks to patients and yet offer hope of therapeutic efficacy. A number of these protocols are described in the supplementary material. Of necessity, such combinations are speculative, and though the evidence for the individual agents may be strong, the evidence for these combinations is often mechanistic or based on pre-clinical data only. While there is a need for more pre-clinical studies, it can be argued that given the urgency of patient need and the low toxicity of these proposed combinations, it is acceptable that small patient trials will begin in the near future.

## Conclusion

The evidence for an anti-cancer effect of CIM treatment comes from *in vitro*, *in vivo*, and considerable amounts of human data. There are a number of well-described mechanisms of action, particularly of multiple immunomodulatory effects which have been assessed in data from clinical trials as well as from *in vivo* models. As an agent CIM has well-established pharmacokinetics and an excellent toxicity profile. Its use in clinical trials together with several chemotherapeutic agents has not shown any clinically relevant interactions, except with epirubicin, and showed evidence of a possible protective effect with vinorelbine and cisplatin. It is, therefore, a very strong candidate for repurposing as an oncological treatment, particularly as a perioperative treatment for surgical resection of solid tumours, in combination with existing standard treatments and alongside other repurposed drugs in a range of cancers.

A number of these multi-drug combinations have been outlined in the supplementary material in the hope that clinicians act upon this data to initiate clinical trials as a matter of some urgency.

## Author contributions

Primary author: Pan Pantziarka. Contributing authors: Gauthier Bouche, Lydie Meheus, Vidula Sukhatme and Vikas P Sukhatme. All authors read and approved the final manuscript.

## Competing interests

The authors declare that they have no competing interests. All the authors are associated with not-for-profit organisations that aim to repurpose drugs for oncology treatments.

## Figures and Tables

**Table 1.** Summary of evidence by cancer type.

Cancer Type	Pre-clinical	Case Report	Clinical Trial
**Colorectal**	[27]		[34–40] ACTRN12609000769280
**Melanoma**	[25]	[42]	[43–46, 48, 50]
**Gastric**	[28]		[54, 55]
**Renal cell carcinoma**			[56, 57, 59–62]
**Prostate**			[65] NCT00684970
**Pancreatic**	[29]	[64]	NCT01509911, NCT01659502,
**Glioblastoma**	[31]		
**Lungs**	[30]	[33]	
**Ovarian**	[26]		[66]

**Table A1.** Proposed drug combinations with CIM for specific indications.

Disease	Targets	Drug Combination
**Malignant melanoma**	Microtubule disruption, inhibition of autophagy, anti-angiogenic, and immunomodulation	Hydroxychloroquine *(NCT00962845)* Diclofenac or Celecoxib [43] Oral cyclophosphamide [44] Mebendazole [45]
**NSCLC**	AMPK/mTOR, Hedgehog signalling, COX-2 inhibition, and immunomodulation	Metformin *(NCT01997775)* Itraconazole [8] Diclofenac or Celecoxib *(NCT00520845)*
**Glioblastoma multiforme**	Inhibition of autophagy, microtubule disruption, Hedgehog pathway inhibition, anti-angiogenic, and immunomodulation	Hydroxychloroquine *(NCT00224978)* Itraconazole Mebendazole [28] *(NCT01729260)*
**Colorectal carcinoma**	Microtubule disruption, AMPK/mTOR, immunomodulation, anti-histamine, COX-2, and immunomodulation	Metformin *(NCT01941953)* Diclofenac Oral vinorelbine [46] Mebendazole [47] Aspirin [38]
**Osteosarcoma/soft-tissue sarcoma**	AMPK/mTOR, IGF-I, Hedgehog pathway inhibition, tumour vascularity, anti-angiogenic, and immunomodulation	Metformin Itraconazole Losartan Oral cyclophosphamide [48]
**Breast Cancer (ER+ invasive ductal carcinoma)**	Microtubule disruption, AMPK/mTOR, anti-angiogenic, and immunomodulation	Metformin *(NCT01929811)* Oral cyclophosphamide and/or oral vinorelbine *(NCT00954135)* Aspirin [40]
**Ovarian carcinoma**	Microtubule disruption, AMPK/mTOR, Hedgehog COX-2, and immunomodulation	Metformin *(NCT02050009)* Itraconazole [9] Diclofenac *(NCT01124435)* Mebendazole [49]
**Renal cell carcinoma**	Anti-angiogenic, COX-2, tumour vascularity, Hedgehog pathway inhibition, and immunomodulation	Celecoxib [50] Losartan [50] Itraconazole [51]
**Pancreatic carcinoma**	Microtubule disruption, Hedgehog pathway inhibition, anti-angiogenic, and immunomodulation	Oral cyclophosphamide *(NCT01509911)* Diclofenac *(NCT01509911)* Mebendazole Itraconazole PSK [25]
**Gastric carcinoma**	COX-2, anti-angiogenic, and immunomodulation	Celecoxib [52] Itraconazole [53]

Note that references to clinical trials or published papers are indicative of trials or case reports where the drug (or analogue) has been used for the specific indication.

## Appendix

### Introduction

The following drugs warrant further investigation in combination with cimetidine (CIM), both in pre-clinical studies and potentially in clinical trials. These combinations, listed in Table A1, have been selected on the basis of existing pre-clinical and clinical experience in each of the indications. In some cases, these combinations replicate existing protocols currently being tested in clinical trials, but substitute known and repurposed drugs for the newer and/or more toxic agents currently being investigated. All these proposed combinations are expected to display relatively low toxicity and use low-cost and generally available agents.

### Higher-priority agents

The agents listed below have a high degree of clinical evidence of efficacy and are currently either in clinical use in oncology or are currently being investigated in clinical trials. They have been selected as potential agents to be used in combination with CIM. Note that these drugs are not listed in order of priority.

- Metronomic chemotherapy—There is increasing clinical interest in using metronomic dosing schedules in a range of existing chemotherapeutic drugs, particularly cyclophosphamide, capecitabine, etoposide, temozolomide, and vinorelbine [1, 2]. At the continuous low doses used in metronomic chemotherapy, there is little evidence of a direct cytotoxic effect on tumour cells, with increasing evidence that the therapeutic effect is primarily driven by anti-angiogenic and immunomodulatory actions [3, 4]. Combining low-dose metronomic chemotherapy with other immunotherapeutic agents is an attractive proposition that is being actively investigated in clinical settings [5, 6]. Given the strong evidence that CIM acts in multiple immunomodulatory ways, there is every reason to believe that it would synergise with existing metronomic chemotherapy regimens to reverse cancer-induced immune suppression, improve activity of cytotoxic T lymphocytes, and prime immune responses in a Th1 direction. There is no evidence to suggest that the addition of CIM to these regimens would increase the lower levels of toxicity associated with metronomic chemotherapy.
- Itraconazole—This broad spectrum antifungal drug is being, or has been, clinically investigated as an anti-cancer agent in a number of trials, including for metastatic prostate cancer (NCT00887458), basal cell carcinoma [7], non-small cell lung cancer [8], refractory ovarian cancer [9], and triple-negative breast cancer [10]. The putative mechanisms of action are anti-angiogenic and inhibition of the Hedgehog signalling pathway [11, 12]. There is a pre-clinical evidence to suggest that the combination of Hedgehog pathway inhibition and the targeting of myeloid-derived suppressor cells (MSDC) may be a beneficial strategy in some hard to treat tumours such as pancreatic cancer [13]. It is suggested, therefore, that the combination of itraconazole and CIM be investigated in pancreatic and other solid tumours. It should be noted that there is some evidence in animal models of an interaction between itraconazole and CIM, such that the AUC of CIM was increased by 25%, an effect which may be of clinical benefit in extending the therapeutic effect of this drug combination [14].
- Diclofenac/Ketorolac—As with a number of other NSAID (particularly those with evidence of COX-2 inhibitory properties), diclofenac shows some evidence of anti-cancer activity. There is evidence that perioperative or intraoperative diclofenac or ketorolac may be associated with lower risk of cancer recurrence or metastatic spread following surgical resection of tumours [15]. Additionally, there is pre-clinical evidence of a direct anti-cancer role of diclofenac in a number of different malignancies. For example, there is *in vivo* evidence in ovarian cancer [16], in melanoma [17], and glioblastoma [18]. Diclofenac has also been used in the treatment of desmoid tumours in adults and paediatric patients, including in combination with vinblastine [19]. Mechanisms of action of diclofenac include anti-inflammatory effects, inhibition of COX-2 and effects on tumour cell metabolism [17]. Of particular interest is the immunological effect of diclofenac, which has been shown, *ex vivo*, to potently reverse post-irradiation immunosuppression [20]. It is hypothesised that effects of diclofenac or ketorolac, including anti-inflammatory and pro- immunity effects, would synergise with the immunotherapeutic effects of CIM and that this combination warrants clinical investigation.
- PSK/PSP—PSK (Polysaccharide K/Krestin) is a protein-bound polysaccharide isolated from the cultured mycelium of the CM-101 strain of the mushroom *Coriolus versicolor*, which has a long history of medicinal use in traditional Chinese medicine. PSP (Polysaccharopeptide) is a related compound from the COV-1 strain of *Coriolus versicolor* that is also used medicinally, although there has been a less clinical investigation of PSP compared to PSK [21]. PSK has been used clinically in Japan since the late 1970s, principally with chemotherapy for the post-operative treatment of gastric, colorectal, and small-cell lung cancers. Numerous clinical trials and meta-analyses have indicated that there is a statistically significant effect on overall and disease-free survival for patients treated with curative resection followed by adjuvant PSK in these three cancers, and these are effectively summarised by Maehara *et al* [22]. There have been fewer clinical trials evaluating the use of PSP, though it is notable that a small trial in non-small cell lung cancer patients found a slower disease progression than in non-treated controls [23]. Also notable is a double-blinded clinical trial in canine hemangiosarcoma which found that high-dose PSP significantly delayed the progression of metastases and afforded the longest survival times reported to date in this condition [24]. The anti-cancer activity of both PSK and PSP is understood to be primarily due to diverse effects on the immune system, including reversal of immune suppression, upregulation of Th1 cytokine production and direct effects on tumour cells. These mechanisms of action parallel those of CIM, suggestive of a possible synergy that should be investigated in particularly intractable malignancies, such as pancreatic cancer [25] or soft-tissue sarcomas.
- Mebendazole—This widely used anthelmintic drug has shown pre-clinical and clinical evidence of activity against a range of cancers. It is being investigated in two clinical trials, with temozolomide, in glioblastoma, and there are case reports in colorectal and adrenocortical carcinoma [26]. The primary mechanism of action as an antiparasitic is the inhibition of tubulin polymerisation in the gut of helminths, and microtubule disruption has also been assessed in relation to its potential anti-cancer activity, for example in a melanoma and glioblastoma models [27, 28]. There is some evidence that CIM increases the peak plasma levels of mebendazole [29], an effect that may prove therapeutically useful in combination protocols. In particular, both drugs show evidence of anti-cancer activity in colorectal cancers, and it is suggested that clinical or pre-clinical investigation for colorectal disease is warranted.
- Hydroxychloroquine—The anti-malarial drugs chloroquine and hydroxychloroquine are known inhibitors of autophagy currently being investigated with a range of standard of care treatments against cancer [30]. The rationale is that cellular stresses generated by cancer treatments induce an autophagic response from tumour cells and that this autophagic state confers resistance to treatment. Inhibition of autophagy in such cases blocks this resistance and increases radio- and chemo-sensitivity [31]. However, there is intriguing evidence that the effectiveness of autophagy inhibition as an effective strategy can be compromised by defective immune responses [32, 33]. Possibly this is because autophagy inhibition leads to greater immunogenic cell death (ICD) in certain treatment modalities, such as radiotherapy, photodynamic therapy and other ROS-related cell death pathways (though not necessarily for certain chemotherapeutic agents) [32, 34, 35], but in an immunosuppressive environment ICD does not lead to greater anti-tumour immune response. The use of CIM to reverse immunosuppression in parallel to autophagy inhibition, (with chloroquine, hydroxychloroquine or other inhibitor, such as the antibiotic clarithromycin [36]), could therefore be a fruitful strategy to pursue in a clinical trial.
- Aspirin—There is abundant epidemiological evidence of a cancer-preventative effect of long-term aspirin usage, summarised for example by Thun *et al* [37], in recent years there has also been an increasing interest in the adjuvant effects of aspirin treatment [38–40]. In particular the use of aspirin post-diagnosis has been shown to be associated with improved overall survival in colorectal, breast, prostate, and oesophago-gastric cancers [40, 41]. There is also some evidence that aspirin use is associated with improved long-term survival in non-small cell lung cancer treated with curative resection [42]. It is proposed, therefore, that aspirin be combined with CIM for long-term post-operative protocols in a number of cancer indications, including colorectal and breast cancer.
